# Parasitoid Insect Venom Proteins: Identification, Functions, Evolution, and Biocontrol Potential—Lessons from Hymenoptera and Open Questions in the Coleopteran Ectoparasitoid *Dastarcus helophoroides*

**DOI:** 10.3390/insects17060608

**Published:** 2026-06-09

**Authors:** Huayang Yin, Qingtong Wang, Zhe Liu, Wanlin Guo, Xiaojuan Li

**Affiliations:** 1 Institute of Forest Protection, Anhui Provincial Academy of Forestry, Hefei 230031, China; yinhuayang211@gmail.com (H.Y.); qtwang@njfu.edu.cn (Q.W.); 2 School of Forestry and Landscape Architecture, Anhui Agricultural University, Hefei 230036, China; liuzhe0916@gmail.com

**Keywords:** parasitoid venom, host manipulation, immune suppression, developmental arrest, metabolic reprogramming, venosome, Hymenoptera, *Dastarcus helophoroides*, biological control

## Abstract

In many parasitoid insects, especially hymenopteran parasitoids, venom is delivered around oviposition to override host physiology. Once viewed as a narrowly immunosuppressive secretion, parasitoid venom is now recognized as a multifunctional effector system targeting host immunity, development, metabolism, and behavior. This review synthesizes the venom apparatus, identification strategies, mechanisms of host manipulation, evolutionary processes, and biocontrol prospects, drawing primarily on Hymenoptera. A consistent pattern emerges: comparable host phenotypes arise from divergent molecular scaffolds, indicating functional rather than molecular convergence. Mechanistic evidence is strongest along six interlocking axes: inhibition of the prophenoloxidase cascade and melanization, disruption of cellular and humoral immunity, developmental arrest, metabolic reprogramming, and neuromuscular or behavioral manipulation. Outside Hymenoptera, the coleopteran ectoparasitoid *Dastarcus helophoroides* offers one of the clearest comparative cases: parasitization inhibits host melanization, alters phenoloxidase activity, and reduces antibacterial activity and circulating hemocyte abundance. As of today, fifty putative venom-like candidate proteins have been identified from larval whole-body proteomics, yet the secretory tissue remains anatomically unresolved, and no individual effector has been functionally validated. The central challenge is therefore the transition from candidate catalogs to mechanistically resolved repertoires, particularly outside Hymenoptera. Closing this gap will be important for evolutionary inference and the rational development of venom-based biocontrol tools.

## Simple Summary

Parasitoid insects are insects whose larvae develop on or inside another insect and eventually kill it. To make the host suitable for their offspring, many parasitoids inject venom-like secretions that weaken host defenses, delay development, alter nutrition, or change movement. This review summarizes what is known about the proteins in these secretions, how they are discovered, how they act in the host, how they evolve, and how they might help biological control. Most detailed knowledge comes from parasitic wasps. By comparison, much less is known about parasitoid beetles. We highlight *Dastarcus helophoroides*, a beetle used against wood-boring longhorned beetles, because host immune suppression and candidate venom-like proteins have been reported, but the source tissue and the function of individual proteins remain unknown. We argue that the next step is to move from lists of candidate proteins to experiments that identify the active molecules and their host targets, which is also a prerequisite for any rational use of these molecules in environmentally friendly pest management.

## 1. Introduction

Parasitoid insects, most of them Hymenoptera, complete larval development on or within another arthropod host. In hymenopteran systems, successful parasitism usually hinges on rapid modification of host physiology at or around oviposition. This manipulation is achieved through maternal factors delivered with the egg, including venom, polydnaviruses (PDVs), virus-like particles (VLPs), and ovarian proteins, and by later offspring-derived products such as teratocyte secretions [1,2,3]. Among these components, venom is often among the earliest-acting and, in many systems, provides the most tractable molecular interface between parasitoid and host.

Parasitoid venoms were long treated as immune suppressants, and the emphasis was justified: parasitoid eggs and larvae, particularly those of endoparasitoids, must survive host encapsulation, melanization, hemocyte attack, and antimicrobial responses [4]. Their functional scope, however, extends well beyond immune evasion. Experimental studies now show that venom proteins act along six interlocking axes: they block melanization and the prophenoloxidase (proPO) cascade, dismantle cellular immunity, suppress humoral immune signaling, arrest host development, redirect metabolic flux, and hijack neuromuscular or behavioral output [5,6,7,8,9,10,11]. Recent syntheses accordingly treat parasitoid venom as a general host-manipulation system rather than a narrowly immunosuppressive secretion [3,12].

The evidence base behind this conceptual shift remains taxonomically uneven. Mechanistic work is concentrated in hymenopteran systems such as *Nasonia vitripennis*, *Pteromalus puparum*, *Leptopilina* spp., *Microplitis mediator*, *Cotesia* spp., *Habrobracon hebetor*, *Pachycrepoideus vindemmiae*, and *Sclerodermus guani* (often cited in earlier and applied literature as *Scleroderma guani*) [13,14,15,16,17], whereas non-hymenopteran parasitoids remain largely unexplored. The coleopteran ectoparasitoid *Dastarcus helophoroides* offers one of the clearest currently available comparative cases outside Hymenoptera: larval parasitization of its cerambycid hosts inhibits melanization, alters phenoloxidase (PO) activity, lowers antibacterial activity, and reduces circulating hemocyte numbers [18], and proteomic analysis of whole-body extracts from neonate larvae has identified 50 candidate venom-like proteins [19]. Because these candidates were inferred from larval whole-body proteomics rather than from anatomically verified secretory tissue, they should be treated as putative venom-like factors until secretion and delivery are demonstrated. The secretory tissue itself has not yet been anatomically resolved, and no individual effector has been functionally tested; these gaps are revisited in detail in Section 6.

This asymmetry shapes our approach. We use the hymenopteran literature as the mechanistic core of the review and treat *D. helophoroides* as an emerging comparative model whose current limitations are themselves instructive. Across the sections that follow, we examine the composition of the venom apparatus and its secreted repertoire, the strategies for identifying venom proteins and their associated biases, the mechanisms by which venom proteins act on host immunity, development, metabolism, neuromuscular function, and behavior, the evolutionary processes that shape venom repertoires, a dedicated comparison of the coleopteran ectoparasitoid *D. helophoroides* against this hymenopteran framework, and the findings sufficiently mature to inform applied discussion in biological control. Throughout, we treat parasitoid venoms as rapidly evolving, functionally convergent effector systems shaped by lineage history, parasitism mode, and host biology. Unlike recent syntheses that broadly address parasitoid-derived host manipulation factors [3,12], this review focuses specifically on venom proteins, the evidence hierarchy linking candidate discovery to functional validation, and the non-hymenopteran comparison provided by *D. helophoroides*. This is a narrative rather than a systematic review; we prioritized peer-reviewed studies that link venom-gland or venom-proteomic discovery to host phenotypes or functional validation, while treating *D. helophoroides* as a focused non-hymenopteran comparison because it currently provides host-level immune data together with a preliminary candidate-protein catalog.

## 2. The Venom System: Apparatus and Composition

### 2.1. Architecture of the Venom Apparatus

**Figure 1:**
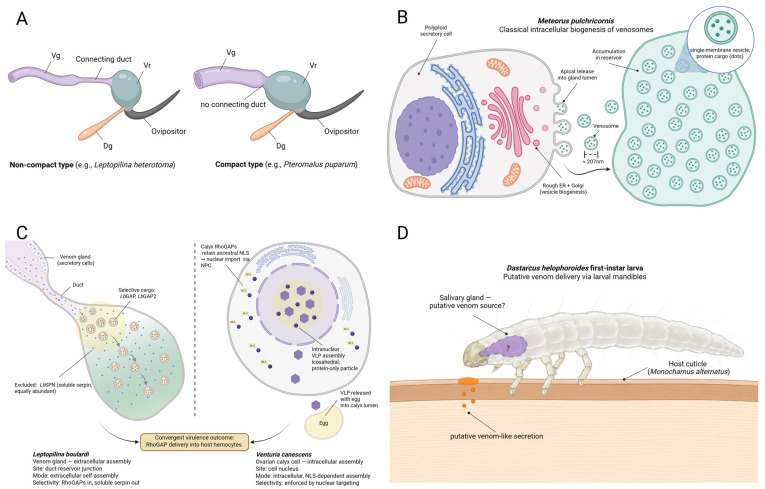
Architecture of the parasitoid venom apparatus and the extracellular vesicles that package venom effectors. (**A**) Comparative anatomy of two hymenopteran venom apparatuses. The figitid *Leptopilina heterotoma* carries an elongated “long gland–reservoir” complex lined with large polyploid secretory cells, contrasting with the more compact gland–reservoir arrangement typical of pteromalids such as *Pteromalus puparum*. Both systems deliver venom through the ovipositor during oviposition. Vg, venom gland; Vr, venom reservoir; Dg, Dufour’s gland. (**B**) Ultrastructural origin of venosomes in *Meteorus pulchricornis*: single-membrane vesicles of ~207 nm associated with secretory-cell compartments in polyploid secretory cells accumulate in the gland lumen and reservoir. (**C**) Two contrasting biogenesis routes for venom-associated extracellular vesicles. In *Leptopilina boulardi*, venosomes (100–300 nm) assemble extracellularly at the junction between the gland’s connecting duct and the reservoir; they selectively package the Rho GTPase-activating proteins (RhoGAPs) LbGAP (the *L. boulardi* RhoGAP) and its paralog LbGAP2 (~87% of LbGAP is vesicle-associated), while excluding the equally abundant soluble serpin LbSPN. In *Venturia canescens*, virus-like particles (VLPs) form intracellularly inside ovarian calyx cells; calyx RhoGAPs retain an ancestral nuclear-localization signal (NLS) that mediates their import through nuclear pore complexes (NPCs) and is required for intranuclear VLP assembly. Despite their different organs and biogenesis routes, venosomes and VLPs converge on the same virulence outcome: delivery of RhoGAPs into host hemocytes. (**D**) Unlike hymenopteran females that inject venom through an ovipositor, the coleopteran ectoparasitoid *Dastarcus helophoroides* interacts with its cerambycid host (*Monochamus alternatus*) during external larval feeding; salivary or other larval secretory tissues have been proposed as possible sources of host-modifying secretions, but the secretory tissue and delivery route remain unresolved (dashed outline, question mark). Figure created by the authors based on evidence from [18,19,20,21,22,23].

In hymenopteran parasitoids, venom is synthesized by secretory cells of the venom gland, stored in a reservoir, and delivered into the host through the ovipositor at oviposition. Although this arrangement is broadly conserved, the anatomy and cellular organization of the apparatus vary substantially among taxa. In *Leptopilina heterotoma*, the gland-reservoir–ovipositor complex forms an elongated “long gland–reservoir” organ lined with large polyploid secretory cells [20], contrasting with the more compact arrangements typical of many chalcidoids and braconids (Figure 1A). In eupelmid egg parasitoids such as *Anastatus japonicus*, *A. fulloi*, *Mesocomys trabalae*, and *M. albitarsis*, the gland–reservoir complex enlarges with female age and reaches maximum size 6–7 days after emergence, indicating that apparatus maturation is developmentally dynamic rather than static [21].

Ultrastructural studies have added a mechanistic dimension to this morphological framework. Transmission electron microscopy of *Meteorus pulchricornis* identified venosomes, single-membrane vesicles of ~207 nm that arise in secretory cells and accumulate in the gland lumen and reservoir (Figure 1B) [22]. Subsequent proteomic work on extracellular vesicle (EV)-like venom fractions in *Leptopilina* and related systems has reinforced this picture [23,24]. Parasitoid venom, at least in some taxa, is therefore not delivered as a simple soluble protein mixture; part of the biologically relevant unit is a protein packaged within a vesicular carrier.

The apparatus must also be interpreted within the broader maternal parasitism arsenal. Venom is not equally important across all parasitoids. In the PDV-associated ichneumonid *Hyposoter didymator*, venom gland extract is dispensable for successful parasitism, and host alteration is attributable mainly to ovarian fluid and viral factors [25]. In *Cotesia typhae*, by contrast, neither venom nor ovarian fluid alone restores full parasitism success in a weakly virulent strain, whereas the combination does [26]. Venom is therefore not universally sufficient; in virus-associated lineages especially, it is best viewed as one component of an integrated maternal secretion system.

### 2.2. Compositional Landscape: Shared Scaffolds and Lineage-Specific Effectors

Transcriptomic and proteomic studies have cataloged venom repertoires across a broad range of parasitoid wasps, with the number of identified candidates ranging from 16 in *Aphidius ervi* to over 200 in *Habrobracon hebetor* [27,28]. Despite this variation, several protein families recur across distantly related taxa and constitute what appears to be a shared structural scaffold of parasitoid venoms. The recurrent families include serine proteases and their serpin-, Kunitz-, and Kazal-type inhibitors [29,30,31]; M12 reprolysin-like metalloproteases [15,32,33,34]; hydrolases such as lipases, esterases, glycosyl hydrolases, and acid phosphatases [29,31,35]; calreticulin [16,32,36,37]; and oxidoreductases including superoxide dismutase [15,32,38].

Several of the most consequential venom activities are nevertheless mediated by lineage-restricted proteins rather than by these universally conserved families. Rho GTPase-activating protein (RhoGAP)-domain proteins in *Leptopilina*, including LbGAP and its paralogs, are major virulence factors that interfere with cytoskeletal signaling in host hemocytes [39,40]. In *A. ervi*, gamma-glutamyl transpeptidases dominate the identified venom fraction and have been linked to reproductive and nutritional effects on aphid hosts [27,41]. DUF4803-domain proteins in *Asobara japonica* and related braconids trigger imaginal disc degradation and delay host development [9,11]. In *Ganaspis*, a venom-specific SERCA calcium pump blocks the calcium burst required for plasmatocyte activation [42,43]. The jewel wasp *Ampulex compressa* is more distinctive still: its venome, defined as the catalog of proteins recovered from the venom apparatus and its secreted output, is enriched in metalloproteases, phospholipases, adenosine deaminase, neuropeptide precursors, and amphipathic peptides specialized for manipulation of the host central nervous system rather than for the immune suppression characteristic of reproductive parasitoids [44].

Many venom repertoires also contain taxonomically restricted or poorly annotated proteins with no detectable homologs outside narrow clades, a feature emphasized in genome-enabled systems such as *Nasonia vitripennis* [45,46]. The mixture of recurrent scaffolds and lineage-specific innovations helps explain why broadly similar host phenotypes can emerge from compositionally divergent venoms.

The coleopteran case offers an informative but provisional contrast. Isobaric tags for relative and absolute quantitation (iTRAQ)-based proteomics of *D. helophoroides* neonate larvae reported 50 putative venom-like or parasitism-associated candidate proteins (19 arginine kinases, 10 chitinases, and 21 proteases or protease inhibitors), with the salivary gland proposed as a possible source of venom-like secretions on the basis of the larva’s mouthpart-mediated attack on the host [19]. Some of these classes overlap with hymenopteran venoms, but the prominence of arginine kinases is unusual. Because the analyzed material consisted of whole-body extracts rather than a dissected and anatomically verified gland, it remains unclear whether this profile reflects a genuine coleopteran venom signature or a mixture of secreted effectors and non-venom contaminants.

## 3. Identification Strategies for Venom Proteins

### 3.1. From Classical Biochemistry to Venom Gene Discovery

Early work on parasitoid venoms relied on targeted purification, molecular cloning, and recombinant expression of individual proteins. The recombinant venom protein rVPr1 from *Pimpla hypochondriaca* exemplifies this approach: it was isolated and characterized as a factor that suppresses host hemocyte activity and increases susceptibility to entomopathogens [47]. Classical methods retain their value because they can establish a direct link between molecular identity and biological function. Their drawback is throughput: they are well suited to dissecting single proteins but poorly suited to resolving the complexity of an entire venom mixture.

### 3.2. Venom Gland Transcriptomics

High-throughput sequencing of the venom gland has become the most common entry point for venom discovery. In its simplest form, transcriptome-based candidate identification applies three criteria: strong expression in venom gland tissue relative to non-venom tissues, an N-terminal signal peptide consistent with secretion, and homology to known venom-associated proteins or domains [37,45]. In practice, the third criterion is often less informative than expected, because many venom genes are lineage-specific or highly diverged.

Transcriptomics has proved especially useful in non-model species for which genomic resources remain limited. Venom gland datasets from *Cotesia vestalis* (457 putative venom genes), *Diadromus collaris* (532), *Gonatopus flavifemur* (154 venom-gland-associated genes), *Diversinervus elegans* (48 putative venom proteins, identified by an integrated transcriptomic-proteomic approach), and *H. hebetor* (34 putative venom proteins from the venom-gland transcriptome) have substantially expanded the taxonomic breadth of the field and generated prioritized candidate lists for downstream work. These counts are not directly comparable, however, as the studies defined venom candidates differently—from predicted secretory proteins to differentially expressed venom-gland genes [37,48,49,50]. The *N. vitripennis* transcriptome further underscored a broader methodological point: novel and poorly conserved sequences contribute substantially to venom repertoires, so annotation pipelines that rely heavily on homology risk overlooking biologically important effectors [45]. By contrast, no venom-gland or secretory-tissue transcriptome is yet available for the coleopteran ectoparasitoid *D. helophoroides*; its molecular inventory still rests entirely on whole-body proteomics of neonate larvae [19], and transcriptomic analysis of its secretory tissue has yet to be undertaken. Such a dataset will be needed before the species can be compared directly with hymenopteran venom systems (Section 6).

### 3.3. Proteomics

Proteomics provides the strongest direct evidence that a given protein is physically present in the secreted venom mixture. Early liquid chromatography–tandem mass spectrometry (LC-MS/MS) studies of *P. puparum* and *N. vitripennis* established the feasibility of coupling protein identification with venom reservoir dissection, moving the field beyond transcript-based inference [15,29,51]. Quantitative proteomics has since been applied not only to catalog venom proteins but also to compare venoms among species, strains, and sampling methods.

The choice of collection method materially shapes the apparent venom profile. In *H. hebetor*, proteins recovered from dissected reservoirs overlapped only partially with those obtained from artificial hosts (25 shared proteins among 148 reservoir proteins), illustrating how strongly the sampling strategy can bias the picture [28]. The issue is especially relevant for understudied taxa. In *D. helophoroides*, the current candidate set was generated from whole-body extracts of neonate larvae because their small body size precluded salivary gland dissection; the salivary gland is only a hypothesized, not a verified, venom reservoir [19]. The resulting proteomic inventory is informative but lacks the anatomical precision expected in hymenopteran venomics.

### 3.4. Integrated Multi-Omics

The most reliable discovery pipelines combine proteomic and transcriptomic evidence. A candidate carries substantially more weight when it is both detected in the secreted material and encoded by a transcript enriched in the venom gland. This logic underpins work on *Chelonus inanitus*, *Cotesia chilonis*, *Toxoneuron nigriceps*, *Pachycrepoideus vindemmiae*, and *Cotesia congregata* [16,17,32,52,53], and it reduces the risk of misassigning abundant housekeeping proteins to venom function.

Multi-omics is now moving beyond simple transcript–protein intersection. In *Leptopilina*, an integrated genomic, proteomic, and biochemical analysis identified a genus-conserved venom lipase, the *Leptopilina*-specific venom lipase (LVL), whose pH optimum (pH 6–7) is co-adapted to the slightly acidic host hemolymph induced during parasitization [54]. Such integrated multi-omics designs not only improve candidate identification but also reveal functional categories that a single omics layer would miss.

### 3.5. Candidate Prioritization and Current Bottlenecks

Expression level alone is insufficient to classify a protein as a venom effector. Venom glands contain secretory proteins, structural proteins, metabolic enzymes, and transcripts unrelated to parasitic virulence. Current prioritization pipelines therefore combine signal-peptide prediction, proteomic support, functional domain annotation, cross-species comparison, and, where possible, an explicit host phenotype [30,46]. Such filtering is necessary but does not address the field’s central difficulty.

The principal bottleneck in parasitoid venomics is the gap between candidate identification and functional validation. Omics studies have generated thousands of candidates across taxa, yet only a small minority have been linked to specific mechanisms through recombinant protein assays, RNA interference (RNAi), transgenic expression, or direct biochemical interaction analysis [17,46,55]. The imbalance constrains biological interpretation at every level, from comparative evolution to applied discussion, and is particularly acute in emerging systems such as *D. helophoroides*. A comparative summary of current collection strategies, their typical yields, and their principal biases is provided in Table 1.

### 3.6. Beyond Identification: Tools for Functional Validation

Closing the gap between candidate identification and mechanistic resolution requires a different toolkit from the one used for discovery. RNAi has proved feasible in several systems, including *M. mediator* and *A. ervi* [41,56,57], and remains the most accessible loss-of-function approach in non-model parasitoids. Transgenic expression in *D. melanogaster* has provided a useful heterologous platform for venom proteins from *Pachycrepoideus*, including PvKazal and PvCRT [36,58]. Recombinant protein assays remain indispensable for establishing direct biochemical activity on host substrates, and metabolomics has become increasingly informative for phenotypes that immune assays alone would miss [5,10].

Three further developments are likely to be particularly influential over the next decade. Single-cell or spatial transcriptomics could resolve secretory cell heterogeneity within the venom apparatus, which is largely invisible in bulk RNA-seq. Genome editing in the parasitoid itself would permit direct loss-of-function tests in the source organism rather than only in the host. Structural characterization of venom protein–target complexes would sharpen mechanistic inference for multifunctional families such as serpins, metalloproteases, and lipases, where biochemical assays alone cannot distinguish among alternative substrate interactions. The emerging functional toolkit will be essential to convert the descriptive catalogs built in Section 3.1, Section 3.2, Section 3.3, Section 3.4 and Section 3.5 into the mechanistically resolved repertoires synthesized in Section 4.

## 4. Functional Roles of Venom Proteins in Host Manipulation

### 4.1. Immune Suppression

Immune suppression is the best-resolved area of parasitoid venom biology. Across Section 4.1, Section 4.2, Section 4.3 and Section 4.4, we organize the evidence around six functional axes: three within immune suppression (the proPO cascade and melanization, cellular immunity, and humoral immunity) and three further axes acting on host development, metabolism, and neuromuscular or behavioral output. Within Section 4.1, the strongest mechanistic evidence falls into three categories: inhibition of the prophenoloxidase (proPO) cascade and melanization, disruption of cellular immunity, and suppression of humoral immune signaling. The functional repertoire of parasitoid venom across all six host-manipulation axes discussed in this section, together with mechanistically validated effectors and their identified host targets, is cataloged in Table 2.

#### 4.1.1. Inhibition of the proPO Cascade and Melanization

Multiple parasitoid lineages have independently recruited venom proteins that block the serine protease cascades leading to phenoloxidase activation. In *P. puparum*, PpS1V, one of 16 alternatively spliced isoforms of *PpSerpin-1*, forms sodium dodecyl sulfate (SDS)-stable covalent complexes with two host clip-domain serine proteases, PrPAP1 and PrHP8 (stoichiometry of inhibition SI ≈ 2.3 mol PpS1V per mol target protease), and cross-reacts with the orthologous clip-SP OfSP13 in the non-natural host *Ostrinia furnacalis*, blocking proPO activation at the proteolytic step without affecting already-activated phenoloxidase [59]. In *M. mediator*, two venom serpins (MmvSPN-1 and MmvSPN-2) form covalent “suicide-substrate” complexes (SDS-stable at ~110 kDa) with the host clip-domain protease HacSP29, thereby disabling the *H. armigera* proPO-activating and Toll-activating cascades; RNAi knockdown of either serpin de-represses both proPO activation and antimicrobial peptide (AMP) synthesis [56]. In *Cotesia rubecula*, the non-catalytic serine protease homolog (SPH) Vn50, whose active-site serine has been evolutionarily replaced by glycine, remains stably uncleaved in host hemolymph and competitively binds proPAP, PAP, proPO, and PO through an intact clip-protease-like domain pair; individual domains, or a mixture of separated domains, show no inhibitory activity, indicating that suppression of proPO activation requires full-length Vn50 to out-compete host SPHs at the proPO interface [60]. In *Leptopilina boulardi*, the 54 kDa venom serpin LbSPNy is the most abundant protein in ISy-strain venom and inhibits proPO activation through classical reactive-center-loop complex formation with host hemolymph serine proteases. Notably, LbSPNy abundance evolves rapidly and host-specifically when *L. boulardi* lines are experimentally maintained on different *Drosophila* species, providing one of the clearest examples of host-driven venom evolution at a single-gene level [61,62].

Other taxa achieve comparable outcomes through different molecular scaffolds. In the aphid parasitoid *A. ervi*, venom interferes with the host proPO-activating cascade at two successive nodes: AeSPH silencing de-represses the upstream proPO-activating factor *PAF2* and reduces mummification by ~60% [57], while recombinant AeSPH1 and AeSPN1 selectively suppress transcription of *ApPPO1* (but not *ApPPO2*), and AeSPH1 RNAi independently lowers mummification by ~55% [41]. In the pupal ectoparasitoid *Pachycrepoideus vindemmiae*, PvKazal inhibits melanization in *Drosophila melanogaster*, an activity confirmed by both recombinant protein injection and transgenic expression [58]. Melanization inhibition is therefore a recurrent functional requirement that parasitoids satisfy through independently recruited protein architectures.

#### 4.1.2. Disruption of Cellular Immunity

Venom-mediated interference with host blood cells is equally well documented. In *Leptopilina boulardi*, the RhoGAP protein LbGAP specifically inactivates host Rac1 and Rac2 GTPases, as verified by yeast two-hybrid assays using constitutively active (G12V) forms and by in vitro GAP activity measurements, and triggers dose-dependent morphological alteration of *Drosophila* lamellocytes. The avirulent ISy strain produces an 89%–identical, functionally equivalent paralog (LbGAPy), but at ~60-fold lower protein abundance than ISm LbGAP. In first-filial-generation (F1) hybrids, almost exclusive expression of the virulent *LbGAP* allele restores full parasitism success, demonstrating that intraspecific variation in virulence is driven by a regulatory threshold rather than by sequence-level differences [39]. The closely related *L. heterotoma*–*L. boulardi* pair illustrates how different immune-evasion strategies can evolve within a narrow phylogenetic frame. In *L. heterotoma*, Lar localizes to the host lymph gland within 12 h of parasitization and triggers apoptotic lysis by 24 h, producing near-complete depletion of circulating lamellocytes; RNAi of *Lar* collapses parasitoid offspring emergence from 77% to 27%. In *L. boulardi*, Warm attaches over 95% of wasp eggs to internal host tissues (mainly the gut) within 4 h of oviposition and provides passive physical protection against encapsulation; RNAi of *Warm* significantly reduces both egg attachment and parasitism success. Both genes carry molecular signatures of lateral gene transfer: *Lar* is embedded within the first intron of *RRP8* and has expanded to 94 genomic loci, while the mucin-bd domain that defines *Warm* is found in 97.9% of bacterial sequences across InterPro. Together, these features are consistent with LGT followed by duplication and neofunctionalization [63].

*M. mediator* deploys at least two distinct venom proteins against cellular immunity. The metalloprotease zymogen VRF1 is cleaved between Arg150 and Val151 to release a 45 kDa C-terminal catalytic fragment (Zn^2+^/Mg^2+^-dependent, K_m_ ≈ 4.3 μM) that localizes near the host-cell nucleus within 24 h and cleaves the NF-κB factor Dorsal at the conserved Cys73/Glu74 site in its Rel-homology domain; in vivo, VRF1 suppresses six antimicrobial peptides (cecropin7, moricin1, moricin5, defensin, gloverin2, lysozyme1) following *Beauveria bassiana* challenge, although the mechanism by which the fragment enters host hemocytes was not resolved [64]. A second RhoGAP, MmGAP1, retains the canonical Arg413 arginine finger and selectively inactivates host RhoA and Cdc42 (with little activity on Rac1), disrupting the F-actin cytoskeleton of host hemocytes. In functional tests, RNAi of *MmGAP1* restores egg encapsulation in 43.1% of host larvae, and microinjection of an anti-MmGAP1 antibody raises encapsulation from 1.5% to 37.5%. These complementary loss-of-function results place MmGAP1 on the causal path from venom delivery to impaired cellular immunity [65]. In *Tetrastichus brontispae*, the acyl-activating enzyme Tb4CL4-like inhibits hemocyte spreading, phagocytosis, and cellular encapsulation [66].

Vesicle-assisted delivery adds a further mechanistic layer. In *Leptopilina boulardi*, atypical extracellular vesicles (venosomes, 100–300 nm) assemble extracellularly at the junction between the gland’s connecting duct and the reservoir (Figure 1C, left), a biogenesis distinct from classical exosomes, microvesicles, and intracellularly formed virus-like particles (Figure 1C, right). Venosomes selectively package the RhoGAPs LbGAP and LbGAP2 (~87% of LbGAP localized to venosomes by immunogold electron microscopy) while leaving the equally abundant serpin LbSPN in the soluble fraction. Functionally, injection of purified venosomes alone is sufficient to protect *L. boulardi* eggs from encapsulation. Their uptake into host lamellocytes is also markedly more efficient in *D. melanogaster* and *D. simulans* (the permissive hosts) than in *D. yakuba* or *D. suzukii*, tracking parasitic success [23]. Recent proteomics of venom-associated extracellular vesicles in *L. boulardi* and *L. heterotoma* recovered ~400 cargo proteins per species, which were enriched in exosomal and endoplasmic reticulum (ER)–Golgi components and included numerous previously unannotated proteins with structural similarity to bacterial ADP-ribosyltransferase toxins, reinforcing the view that part of the functionally relevant toxin package is a vesicle-bound complex rather than a freely soluble protein [24]. Preprint evidence from *Ganaspis hookeri* points in the same direction, showing that venom SERCA itself is packaged in lipid-bound extracellular vesicles that internalize into *Drosophila* plasmatocytes, a plausible transport solution for a large hydrophobic protein that would otherwise be difficult to deliver into host immune cells [43].

The ectoparasitoid *P. vindemmiae* offers a complementary mechanism. Its venom triggers extensive apoptosis in host plasmatocytes and lamellocytes (terminal deoxynucleotidyl transferase dUTP nick end labeling [TUNEL]-positive cells rising from 2% to 98%), only partially blocked by a pan-caspase inhibitor, indicating that caspase-dependent apoptosis operates alongside additional caspase-independent death pathways [69]. Its calreticulin PvCRT further suppresses self-encapsulation when ectopically expressed in *Drosophila* [36], demonstrating that venom factors can inhibit cellular immunity without necessarily killing hemocytes outright.

#### 4.1.3. Suppression of Humoral Immunity

The strongest evidence for humoral immune suppression comes from *P. puparum* and *H. hebetor*. *P. puparum* venom downregulates the host C-type lectin Pr-CTL in a time- and dose-dependent manner, with maximal suppression over the first 8 h post-injection. Recombinant Pr-CTL binds directly to the *P. puparum* egg surface and mediates Ca^2+^-dependent agglutination of mannose, galactose, and peptidoglycan; *Pr-CTL* RNAi in turn reduces cecropin, lysozyme, PAP, and scavenger-receptor transcripts and lowers encapsulation, phagocytosis, PO activity, and antimicrobial activity, identifying Pr-CTL as an upstream amplifying node whose venom-mediated silencing simultaneously removes a host recognition signal and dampens multiple downstream immune effectors [67]. Venom further reduces host cecropin and lysozyme expression in a time- and dose-dependent manner, with inhibition strongest in the first 8 h post-injection [70]. A single venom injection into *Pieris rapae* represses 113 hemocyte transcripts and 221 fat-body transcripts within 1 h. The affected set is enriched for immune function and spans three target categories: upstream activators of the proPO cascade (prophenoloxidase-activating enzymes [PPAEs], hemolymph proteases, and a serpin), but not *proPO* itself; at least ten antimicrobial peptides (including cecropin, lysozyme, attacin, lebocin, proline-rich AMP, gallerimycin, and immune-inducible peptide); and the non-self-recognition receptors Pr-CTL, Gram-negative binding protein (GNBP), scavenger receptor class C (SR-C), and calreticulin [51].

*H. hebetor* venom halves capsule melanization, cuts hemolymph PO activity to one-third of controls by day 2, reduces reactive oxygen species production, and lowers hemocyte spreading capacity from 67% to 33% within an hour of injection in *Galleria mellonella* [71,72]. These immune changes carry downstream ecological consequences: envenomated hosts undergo a dysbiotic collapse of the midgut microbiota, in which operational-taxonomic-unit diversity falls from 54 ± 2.9 to 13 ± 4.6 while *Enterobacter* colony-forming units (CFUs) expand 42- to 45-fold and *Enterococcus* CFUs 10- to 12-fold, and become ~7.5-fold more susceptible to topical *Beauveria bassiana* infection (mortality 12% → 90%), with the antifungal peptide gloverin upregulated 2900-fold under combined venom + fungus challenge. Midgut AMP prioritization and hemocoel encapsulation loss together constitute an antagonistic cross-regulation in the three-component host × parasitoid × fungus system [73]. Across these systems, parasitoid venom therefore suppresses host immunity both locally at specific pathway nodes and globally at the transcriptomic and physiological level.

A comparable host-level immune phenotype is evident in the coleopteran system. Larval parasitization of *Monochamus alternatus* by *D. helophoroides* rapidly inhibits hemolymph melanization, modulates PO activity in a time-dependent manner, lowers antibacterial activity, and progressively reduces total hemocyte counts [18]. These responses point strongly to venom-like immune suppression, but the responsible molecules remain unidentified (examined in detail in Section 6).

### 4.2. Developmental Arrest and Developmental Reprogramming

Parasitoid venoms also interfere directly with host development. The clearest recent example is *Asobara japonica*, in which the DUF4803-domain proteins IDDF-1 and IDDF-2 cooperatively drive host imaginal disc degradation through apoptosis and autophagy, with IDDF-1 additionally required for mitotic arrest. Co-injection of recombinant IDDF-1 and IDDF-2 reproduces only a mild version of the apoptosis/autophagy phenotype and does not elicit mitotic arrest, implying that additional venom components contribute to mitotic arrest and to the full venom-induced phenotype [9]. A parallel set of five DUF4803-domain venom proteins, VID-1 to VID-5, arose by lineage-specific duplication within Braconidae (*A. japonica* retains 91 DUF4803 paralogs, 39 of which have been recruited into venom) and is expressed exclusively in the venom gland. RNAi of any single *VID* collapses host imaginal disc degradation from 100% (60/60) to as low as 3.3% (2/60) without altering the other four paralogs, demonstrating that the five effectors act jointly rather than redundantly. Disc destruction elevates *dilp8*, the top-ranked upregulated gene in eye-antennal discs and the second-ranked in wing discs, and delays host pupation by ~26 h, enabling *A. japonica* to exploit third-instar hosts on which its sympatric competitor *L. drosophilae* achieves only 0.4–4% emergence at early exposures because of host defensive rolling, while *L. drosophilae* retains dominance on younger (second-instar) hosts via faster host location (≈21 s vs. ≈69 s); the VID system thus drives temporal niche partitioning rather than a host shift [11]. Developmental arrest in this system is not a by-product of generalized host sickness but a targeted ecological strategy.

Earlier work pointed in the same direction. Fractionated venom of the ectoparasitoid *Eulophus pennicornis* contains at least two activities with distinct endocrine-like effects: one blocks larval–larval ecdysis, the other produces pupal abnormalities reminiscent of juvenile hormone disruption [74]. In *N. vitripennis*, venom halts chitin biosynthesis, producing 100- to 2072-fold reductions in cuticular-protein transcripts and a 568.7-fold decrease in peritrophin-A, and drives a highly selective shift in host gene expression that affects only ~2% of host loci yet upregulates the Enhancer of split complex (E(spl)-C) by 2.6- to 6.5-fold across all nine *m*/*mα*/*mβ*/*mδ* members, peaking at 24–36 h; the authors propose that a venom metalloprotease cleaves host Notch to release the intracellular domain and activate this cascade [68]. Because these transcriptomic effects co-occur with extensive metabolic remodeling, developmental arrest and metabolic manipulation are mechanistically intertwined rather than fully separable.

The venom protein IMP-L2 of *S. guani* extends this theme. IMP-L2 modulates insulin/insulin-like growth factor signaling (IIS) and target of rapamycin (TOR) signaling in *Tenebrio molitor*, and RNAi knockdown in the parasitoid shortens host survival after parasitization [75]. The venom protein therefore actively prolongs the useful lifespan of the host for the developing parasitoid offspring. Developmental interference in parasitoid systems thus encompasses not only arrest but also active maintenance of host viability under parasitoid control.

### 4.3. Metabolic Manipulation

Metabolic reprogramming is among the most consequential revisions to the traditional view of parasitoid venom. Venom does more than disable host defenses; in many systems it converts host physiology into a more suitable nutritional environment.

The clearest single-protein example is *Leptopilina*-specific venom lipase (LVL). LVL is the only *Leptopilina*-specific venom protein retained in a syntenic block across all four examined species (*L. boulardi*, *L. drosophilae*, *L. heterotoma*, *L. syphax*), yet its clade carries a signature of positive diversifying selection (aBSREL, *p* = 0.018). It preferentially hydrolyzes short-chain host diacylglycerols and triacylglycerols, with a pH optimum of 6–7 that matches the hemolymph acidification (pH 6.9–7.0) induced by *Leptopilina* parasitization itself; artificially neutralizing the host hemolymph with NaHCO_3_ (≈pH 7.8) abolishes both LVL activity and parasitoid embryo hatching. RNAi of *LVL* reduces egg-to-larva hatching by 31.6–54.2% (*p* < 0.001) without altering oviposition number, host phenoloxidase activity, or hemocyte apoptosis, demonstrating that LVL is dedicated to host-lipid mobilization for parasitoid embryogenesis rather than to host immunity [54]. The combination of cross-species conservation and diversifying selection is consistent with a core, non-redundant metabolic role.

Other systems point to different metabolic targets. The venom protein PvG6PDH of *P. vindemmiae* suppresses host glucose-6-phosphate metabolism and redirects carbohydrate allocation in favor of parasitism [76]. In *P. puparum*, genome-scale analysis of venom-associated lipases identified multiple candidates with incomplete catalytic triads and altered structural features, implying that some venom lipases function as lipid-binding or transport proteins rather than as classical hydrolases [77]. In *S. guani*, venom dipeptidyl peptidase IV (SgVnDPPIV) alters host transcriptional patterns of genes linked to lipid synthesis, detoxification, and ion transport [78].

System-level metabolomics confirms that these examples are not isolated. Time-resolved metabolomics of the *N. vitripennis*–*Sarcophaga bullata* system tracked 249 metabolites over five days and documented a 226-fold increase in sorbitol against a backdrop of stable glucose, a 9.56-fold increase in lactate by day 1 (with hemolymph acidification from day 2), selective suppression of α-ketoglutarate and succinate in the tricarboxylic acid (TCA) cycle, complete arrest of chitin biosynthesis (precursors remaining flat against a 4.85-fold developmental increase in controls), and steady accumulation of ten standard free amino acids, a pattern the authors interpret as active venom-directed host reprogramming rather than a passive consequence of paralysis [5]. A parallel study of *H. hebetor*-envenomated *G. mellonella* detected TCA-cycle suppression, amino acid accumulation, elevated trehalose, reduced glucose, and fat body damage [10]. The recurrence of these signatures across phylogenetically distant parasitoids indicates that metabolic conditioning is a widespread and probably central venom function.

### 4.4. Neuromuscular and Behavioral Manipulation

Paralysis is especially prominent in ectoparasitoids, which typically require rapid host immobilization before or during oviposition. *H. hebetor* venom induces permanent flaccid paralysis in lepidopteran hosts, mediated by paralytic peptide toxins purified and biochemically characterized from the venom gland [6]. Transcriptomic analysis indicates that the major paralytic factor is among the most highly expressed venom genes in the species [37].

*A. compressa* represents a more specialized case. Rather than inducing peripheral paralysis, the jewel wasp produces long-lasting hypokinesia in cockroaches by injecting venom into the brain, with prominent downstream effects on central complex (CX) activity. Chronic electrophysiology resolved three temporal phases in CX firing after envenomation: an initial ~80% suppression within 10 min (6.32 ± 1.65 Hz baseline → 2.89 ± 0.48 Hz), a dopamine-driven rebound coincident with the post-sting grooming window (20–30 min), and a sustained decline that stabilizes at ~60% of baseline over 2 h, paralleled by a 33% reduction in descending-interneuron traffic at the neck connectives and a 60% drop in the slow coxal-depressor leg motoneuron [8]. Earlier work confirmed that peripheral sensory neurons continue to propagate signals normally [7], but CX recordings under visual and nociceptive stimuli revealed that the venom actively desensitizes central sensory integration, with light-ON-evoked CX responses falling by ~80% and light-OFF responses by ~45%, so the “behavioral output without sensory access” reading holds only at the periphery; centrally, both pre-motor drive and stimulus-evoked processing are dampened. Proteomic analysis of the 264-protein *A. compressa* venome matches this functional profile: the venom lacks classical ion-channel toxins and cytolysins and is instead enriched in neuropeptide precursors (tachykinin, corazonin, eclosion hormone, myosuppressin), ampulexins, and M13-family metalloproteases (10% of the proteome); it is stored at pH ≈ 4 and processed to mature bioactive peptides only upon neutralization in the host brain, providing a pH-triggered “prolonged time-release” mechanism, with recombinant tachykinin (AcVTk1) sufficient to recapitulate the short-term elevation of escape threshold [44].

Less elaborate paralytic activities are widespread. *Chelonus inanitus* venom has at least three separable activities, including transient paralysis of older host stages, developmental disruption, and altered hemocyte membrane permeability [79]. A broad ecological trend therefore holds, though not without exceptions: ectoparasitoid venoms tend to emphasize rapid immobilization, whereas endoparasitoid venoms tend to emphasize chronic physiological reprogramming of a living internal host. How far these six functional axes extend beyond Hymenoptera can at present be tested in only one non-hymenopteran system, the coleopteran ectoparasitoid *D. helophoroides*, which we therefore treat separately as a dedicated comparison (Section 6).

## 5. Evolution of Parasitoid Venom Repertoires

### 5.1. Origins of Venom Genes: Co-Option and Novelty

The dominant model for venom gene origin in parasitoids has shifted. A comparative genomic–proteomic survey of four pteromalid wasps (*N. vitripennis*, *N. giraulti*, *Trichomalopsis sarcophagae*, *Urolepis rufipes*) showed that of 53 venom genes recently recruited in the *Nasonia*–*Trichomalopsis* complex, 49% are single-copy with no duplication history and a further 34% arose from ancient duplications predating venom recruitment by >15 Myr; only 17% (nine genes) show evidence of recent duplication, and only four of those are unambiguous duplication-based recruitments. Allele-specific expression in *N. vitripennis* × *N. giraulti* F1 hybrids (ρ = 0.85, *p* < 0.001) further attributed this recruitment to *cis*-regulatory evolution in the venom gland, and 13 of 16 subsequent venom-gene losses in *Nasonia* occurred through transcriptional downregulation rather than coding-sequence degradation [80]. A lineage can therefore remodel its venom repertoire rapidly and reversibly, without the extensive gene duplication characteristic of snake or cone-snail venoms.

Although focused on aculeate/bee venoms rather than parasitoid venoms, broader hymenopteran analyses support an evolutionary sequence of ancient recruitment followed by lineage-specific specialization. A 2023 comparative genomic survey of 32 hymenopteran genomes (29 published and 3 newly sequenced at that time), focused on bees, found that the prevalent bee venom proteins (phospholipase A2, icarapin, and hyaluronidase) are encoded as widely conserved single-copy genes that predate the aculeate stinger by >280 Myr and were already present in the symphytan ancestor. Gene duplication was restricted to a few enzymatic families (venom acid phosphatases expanded to 13 paralogs in parasitoid wasps and up to 10 copies in some bee genera) and to short lineage-specific peptides (the bee-unique Anthophilin1 family containing apamin, MCDP, and tertiapin; a *Bombus*-specific melittin tandem duplication). An alignment-independent protein-space analysis further rejected the “aculeatoxin” hypothesis, showing that the short peptides of bees, wasps, and ants occupy distinct sequence-structure spaces [81]. Co-option is not the only route, however. Transcriptomic and proteomic work on *N. vitripennis* and related taxa shows that taxonomically restricted and poorly conserved genes remain a conspicuous component of parasitoid venoms: roughly 29% (23/79) of *N. vitripennis* venom proteins are orphans with no recognizable homolog, 42% of 183 homologous venom-protein groups across four closely related pteromalids lack detectable homology outside Chalcidoidea, and an independent figitid venomics survey of *Ganaspis sp.1* likewise recovered 37 of 166 (~22%) venom proteins with no NCBI match [45,46,80].

### 5.2. Gene Family Expansion and Lateral Gene Transfer

Gene duplication remains important for certain protein families even where single-copy co-option dominates. Comparative analysis of two parasitoid lineages separated by >200 Myr, namely *Leptopilina* (Figitidae) and *Venturia canescens* (Ichneumonidae), revealed convergent recruitment of RhoGAP multigene families from two independent duplications of the cellular *RacGAP1* gene: up to nine venom RhoGAPs per *Leptopilina* species (9 in *L. boulardi* ISm, 8 in ISy, 3 in *L. heterotoma*, 2 in *L. victoriae*, 1 in *L. clavipes*) and 13 calyx RhoGAPs in *V. canescens*. The *Leptopilina* paralog lost the ancestral coiled-coil and C1 motifs and acquired a secretory signal peptide that routes it to the venom apparatus; the *V. canescens* paralogs retained the ancestral nuclear-localization signal required for intranuclear VLP assembly in the ovarian calyx (see Figure 1C, right). In both lineages, all paralogs except LbmGAP/LbyGAP and VcVLP2 lost the catalytic arginine required for canonical GAP activity, 19 codons in the *Leptopilina* RhoGAPs evolved under positive selection (site-directed mutagenesis of 8 of 19 sites identified 5 as essential for Rac binding), yet all mutated paralogs are transcribed, translated, and packaged into extracellular vesicles, namely venosomes in *Leptopilina*, VLPs in *V. canescens*, consistent with neofunctionalization on as-yet-unidentified host targets rather than pseudogenization [40]. The convergence is striking given that venosomes and VLPs form in different organs and via distinct biogenetic routes, yet serve the same virulence function. Family-level expansion is also evident for metalloproteases in *M. mediator* and *M. pulchricornis* [33,34], and for canonical serine protease inhibitors (Kazal, Pacifastin, and TIL families, organized as tandem arrays of up to five genes per scaffold) in *P. puparum*, where serpin diversity is nevertheless generated by alternative splicing of a single gene into up to sixteen exon 8-swapped isoforms rather than by gene duplication [30].

Lateral gene transfer (LGT) has contributed additional novelty. The proteins Lar and Warm in the *L. heterotoma*–*L. boulardi* system were interpreted as products of LGT followed by duplication and functional specialization, and they now underpin markedly different immune-evasion strategies in the two species [63]. LGT is therefore more than an anecdotal mechanism in parasitoid venom evolution; in some cases it has supplied functionally central virulence factors.

### 5.3. Rapid Evolution Under Host-Driven Selection

Parasitoid venom repertoires can evolve rapidly within species. Population analyses of *L. boulardi* and *L. heterotoma* revealed substantial venom differentiation across modest geographic distances, and multivariate quantitative-trait-differentiation (Q_ST_) analysis indicated a strong contribution of selection rather than neutral drift alone [82]. Experimental evolution studies have extended this finding. When *L. boulardi* lines were crossed and maintained for 9–10 generations on different *Drosophila* host strains and species, venom composition shifted rapidly and in a host-specific manner, implying that particular venom proteins can be favored on one host background and disfavored on another [61,62].

Environmental conditions also matter. In *L. boulardi*, venom composition shifts substantially with rearing temperature in a pattern that broadly parallels temperature-dependent parasitic success [83]. At an even shorter evolutionary scale, two strains of *Tetrastichus brontispae* adapted to different beetle hosts exhibit qualitative and quantitative venom divergence after roughly 40 generations [84]. Venom evolution in parasitoids is therefore dynamic at ecological timescales and should not be treated as a fixed species-level trait.

### 5.4. Functional Convergence Without Transcriptomic Convergence

Rapid evolution is not synonymous with random evolution. Unrelated parasitoids routinely achieve similar host outcomes, particularly melanization suppression and cellular immune disruption, yet recent comparative work shows that these repeated phenotypes do not require global convergence of venom gland transcriptomes. In a study of 19 hymenopteran species, parasitoids that had independently adapted to *Drosophila* hosts showed no greater similarity in overall venom gland transcriptome expression than phylogeny alone would predict; most orthogroups with correlated expression were not among the highly expressed venom genes [85]. This result corroborates a theme already evident in functional studies: parasitoid venoms often converge in phenotype while remaining divergent in specific effectors.

### 5.5. Venom in Relation to Polydnaviruses and Other Maternal Factors

Evolutionary interpretation becomes more complex in parasitoids that deploy PDVs or other virulence factors alongside venom. In *H. didymator*, venom has little measurable effect on its own, and host manipulation is driven largely by ovarian fluid containing the associated PDV [25]. In *C. typhae*, by contrast, venom and ovarian fluid act synergistically, and neither is fully effective alone [26]. In *Microplitis demolitor*, venom, PDV products, and teratocyte factors show little molecular overlap, consistent with functional partitioning rather than redundancy [86]. The evolutionary role of venom therefore cannot be assessed in isolation from other maternal secretions, and comparative studies that ignore this context risk either over- or underestimating the contribution of venom itself. For *D. helophoroides*, by contrast, no PDV- or VLP-like factor has yet been documented; if this remains true after targeted searches, the system could provide a comparatively simple test case for venom-like host manipulation outside Hymenoptera, less confounded by known viral symbionts.

## 6. The Coleopteran Ectoparasitoid *Dastarcus helophoroides*: A Non-Hymenopteran Comparison

Section 2, Section 3, Section 4 and Section 5 drew almost entirely on Hymenoptera. Here we turn to the best-characterized non-hymenopteran case and compare it directly with that hymenopteran picture. *D. helophoroides* is a larval ectoparasitoid of cerambycid beetles, most notably *Monochamus alternatus* [18,19]. Like hymenopteran ectoparasitoids, it develops externally on the host and therefore depends on host immobilization and physiological control, a biology that makes it an attractive comparative system. The system is nevertheless far less resolved than any major hymenopteran model, and the gap is informative in itself: the host-level effects are solid, the molecular catalog is preliminary, and almost nothing has been functionally tested.

### 6.1. Phenotypic and Molecular Evidence to Date

At the phenotypic level, *D. helophoroides* clearly suppresses host immunity, and it does so in much the same way as the hymenopteran venoms described in Section 4.1. After parasitization, hemolymph melanization is rapidly inhibited at early post-parasitization time points; PO activity follows biphasic dynamics, with transient stimulation at 4 h, followed by inhibition at 12 h before returning to baseline; antibacterial activity drops within the first hour; and total hemocyte abundance falls to less than half of control levels by 72 h [18]. The same overall pattern is produced by hymenopteran venoms such as those of *P. puparum* and *H. hebetor* [67,71,72,73], so the coleopteran system arrives at the same immune outcome even though none of its individual effectors has yet been identified.

At the molecular level, whole-body proteomics of neonate larvae has assigned 50 candidate venom-like proteins (19 arginine kinases, 10 chitinases, and 21 proteases or protease inhibitors), with the salivary gland tentatively proposed as a possible source or storage site of venom-like secretions, given that the larva paralyzes and feeds on the host through its mouthparts rather than through an ovipositor (Figure 1D) [19]. Notably, 17 of the 19 arginine kinases are more abundant in neonates than in late-stage larvae, prompting the suggestion that this family contributes disproportionately to early host paralysis. Set against the hymenopteran venoms in Section 2.2 and Section 4, this profile is only partly familiar: proteases and protease inhibitors are recurrent venom families across Hymenoptera, whereas the prominence of arginine kinases has no clear hymenopteran parallel. These proteins should therefore be treated as putative venom-like candidates rather than confirmed venom effectors until secretion from a defined tissue, delivery into the host, and activity on host targets are demonstrated.

### 6.2. The Missing Layers of Evidence

The next layer of evidence is absent, and it is here that the coleopteran case lags furthest behind the hymenopteran systems. The secretory tissue has not been conclusively identified; it remains unclear whether the effectors originate from salivary glands, another larval secretory organ, or multiple sources, and the route of delivery into the host has not been mapped. No gland-resolved transcriptome, recombinant validation, or RNAi-based loss-of-function analysis has yet been reported—the same tools that, in Hymenoptera, have turned candidate lists into mechanistically resolved venom repertoires (Section 3.6). *D. helophoroides* should therefore be regarded as a promising but provisional comparative model, one that simultaneously illustrates how much parasitoid venom biology may remain undiscovered outside Hymenoptera and how heavily the interpretation of proteomic data depends on anatomical resolution.

### 6.3. Comparative Predictions from the Hymenopteran Literature

The hymenopteran literature nevertheless offers several testable predictions for *D. helophoroides*, each drawn directly from a hymenopteran precedent. First, the 21 protease and protease-inhibitor candidates should be screened for serpin- and Kazal-class architectures capable of targeting the *M. alternatus* proPO cascade, a hypothesis directly derived from the mechanisms validated in *P. puparum* PpS1V [59], *M. mediator* MmvSPN-1/-2 [56], and *P. vindemmiae* PvKazal [58]. Second, the unusual prominence of arginine kinases, which are rarely abundant in hymenopteran venoms and are disproportionately enriched in neonate larvae, may reflect either a distinct coleopteran solution to early host immobilization, mechanistically separable from the M13-protease/neuropeptide axis exploited by *A. compressa* [44] and from the flaccid paralysis induced by *H. hebetor* venom [6,37], or simply the recovery of abundant non-venom proteins in whole-body proteomics. Distinguishing between these alternatives will require tissue-resolved expression analysis, direct secretion evidence, and loss-of-function assays. Third, the ten chitinase candidates may represent larval secretion- or feeding-associated enzymes involved in host-cuticle degradation or feeding-site modification, an external mode of action that would contrast with the chitin-biosynthesis arrest induced internally by *N. vitripennis* venom [68] and would be consistent with the ectoparasitic life history in which the *D. helophoroides* larva feeds through host integument. Resolving these predictions will require identification of the secretory tissue, a gland-resolved transcriptome, recombinant assays of representative candidates from each functional class, and RNAi targeting the most abundant arginine kinases in neonate larvae.

## 7. Biocontrol Relevance and Future Priorities

### 7.1. Evidence for Applied Potential

Among the most direct evidence for biocontrol relevance comes from studies in which venom proteins enhance other mortality agents or shift competitive outcomes. The recombinant *Pimpla hypochondriaca* venom protein rVPr1 binds host hemocytes, disrupts phagocytosis, and increases susceptibility to both *Beauveria bassiana* and *Bacillus thuringiensis* [47], an early proof of principle that an immunomodulatory parasitoid venom protein can synergize with microbial control agents.

A second applied dimension is ecological. *Ooencyrtus telenomicida* venom impairs the development of the competing egg parasitoid *Trissolcus basalis* within shared host eggs, demonstrating that venom can shape parasitoid community structure as well as host mortality [87]. In the declining *Microctonus hyperodae* biocontrol system in New Zealand, multi-species transcriptomics detected continued expression of at least 36 venom components inside parasitized Argentine stem weevils, including lipase, cathepsin, calreticulin, metalloproteases, and tetraspanins, with no evidence that hosts have evolved venom resistance, since eggs are still not encapsulated; the authors were unable to identify a clear molecular mechanism for the field decline, but discovered a filamentous virus (MhFV) transmitted during both successful and interrupted oviposition attempts, which may underlie a previously observed premature-mortality phenomenon in exposed weevils and could itself be leveraged to restore biocontrol efficacy [88,89].

The coleopteran model *D. helophoroides* adds a complementary, field-scale perspective. Mass-reared releases in Chinese forest management programs have repeatedly achieved high field-level control of *Monochamus alternatus*, a major vector of the pinewood nematode *Bursaphelenchus xylophilus*, which causes pine wilt disease: corrected control rates of up to ~91.48% on felled host trees [90] and corrected population reductions in the 82–86% range have been reported under optimized release ratios and timing [91]. The same parasitoid has been deployed against the related cerambycid *Massicus raddei* in oak forests, where field releases of egg cards yielded a corrected population reduction of 88.6% [92], and against *Anoplophora glabripennis* in urban-forestry settings, although the published case study from Xi’an reported a much lower direct parasitism rate (~8.1% of pharate adults) than the rates documented for conifer-feeding cerambycids [93]. A chromosome-level genome assembly of *D. helophoroides* is now also available to underpin molecular follow-up [94]. Together these studies provide an unusually well-documented operational example in forest pest management, and the gap between this field record and the molecular resolution currently available for the underlying venom-like factors is precisely what makes *D. helophoroides* an informative comparative target.

The most realistic near-term application of parasitoid venom proteins is therefore as host-modifying synergists rather than as standalone broad-spectrum insecticidal toxins. The best-supported use cases are enhancement of host susceptibility to microbial agents, disruption of host developmental timing, and interference with interspecific competition among biocontrol agents.

### 7.2. Translational Constraints: Delivery, Specificity, Resistance, and IPM Positioning

Several constraints separate the proof-of-principle studies above from operational deployment, and each defines an active translational frontier.

The first is delivery. Many functionally characterized parasitoid venom effectors, especially immunomodulatory enzymes and inhibitors, are relatively large proteins that require entry into the host hemocoel or hemocytes to act and are not inherently oral toxins in the way many Bt-derived proteins are. Plausible delivery routes therefore include co-formulation with established microbial control agents (the rVPr1 + *Beauveria bassiana* and rVPr1 + *Bacillus thuringiensis* combinations exemplify this approach [47]), heterologous expression in entomopathogenic fungi or baculoviruses for in-host release, and biomimetic packaging informed by venosome biology [23,24,43]. Each route has unresolved scale-up and stability questions and would require dedicated formulation work before field testing.

The second is host-range specificity and non-target safety. Several validated venom effectors show striking biochemical co-adaptation with their natural host: LVL has a pH optimum (6–7) that matches *Leptopilina*-induced hemolymph acidification [54], and PpS1V cross-reacts with the orthologous clip-SP OfSP13 in the non-natural host *Ostrinia furnacalis* [59]. Such co-adaptation cuts both ways. It can confer natural specificity for a target pest clade, but it can also generate cross-activity on closely related non-target species, including beneficial parasitoids whose own immune cascades share the same molecular architecture. Pre-deployment screening on relevant non-target taxa, including beneficial Hymenoptera and non-target Lepidoptera or Coleoptera depending on the intended pest target, will therefore be a prerequisite for any venom-derived biopesticide.

The third is resistance evolution. As with any single-mode-of-action insecticidal protein, sustained selection pressure could in principle drive resistance in the target pest. The current evidence, although limited, is encouraging: in the *Microctonus hyperodae* system, multi-species transcriptomics found no signature of host-evolved venom resistance even after decades of field decline; eggs continue to escape encapsulation, and the field decline appears instead to involve a previously overlooked filamentous virus (MhFV) [88,89]. Absence of resistance in one system is not a guarantee for others, but the result is consistent with the idea that targeting multiple host nodes simultaneously, as natural parasitoid venoms do, is intrinsically more resistance-resilient than single-target insecticides. This favors, at least conceptually, multi-effector or multi-node strategies rather than reliance on single venom effectors.

The fourth is IPM positioning. Given the constraints above, the realistic near-term role of parasitoid venom proteins is not as standalone broad-spectrum biopesticides but as synergistic components within integrated pest management: enhancers of microbial agents, modifiers of host development to extend windows of biocontrol efficacy, and tools for shifting interspecific competition among parasitoids in mass-rearing or augmentative-release programs [87]. The *D. helophoroides* program in Chinese forest health management, which has already achieved corrected population reductions of *M. alternatus* in the 82–86% range and an 88.6% reduction in *M. raddei* through whole-organism augmentative release [90,91,92], provides a natural platform for the eventual integration of venom-derived molecular tools as a second-generation refinement, once delivery, specificity, and safety barriers are resolved.

### 7.3. Priority Questions for the Next Phase of Parasitoid Venomics

Several priorities follow from the current state of evidence. The first is methodological standardization. Reservoir contents, whole apparatus extracts, artificial-host collections, and whole-body extracts of parasitoid larvae do not yield equivalent proteomic profiles, yet these approaches are often discussed as if they did [19,28]. More consistent venom sampling and reporting would substantially improve cross-study comparability.

The second is integration across phenotype classes. Immune suppression, developmental arrest, metabolic reprogramming, and behavioral manipulation are frequently treated as separate topics, yet many venom proteins act on more than one axis. Progress will be fastest when these phenotypes are investigated jointly rather than in isolation.

The third is taxonomic expansion coupled with anatomical rigor. The field remains strongly hymenopteran-centered. *D. helophoroides* offers one of the clearest current opportunities to test whether current principles of parasitoid venom evolution generalize beyond Hymenoptera. A chromosome-level genome assembly (609 Mb, 14,890 predicted protein-coding genes on 13 chromosomes) is already available [94], so the bottleneck is no longer primarily genomic resources but identification of the secretory tissue, generation of a secretory-tissue-resolved transcriptome, and experimental validation of individual effectors.

The fourth is translation. Proteins such as LVL, PvG6PDH, and IMP-L2 demonstrate that parasitoid venoms can target host nutritional and developmental physiology, not only immunity [54,75,76]. Their applied potential broadens the horizon of the field, but realistic development will require protein engineering, delivery solutions, and careful evaluation of host-range specificity and ecological safety.

## 8. Conclusions

Parasitoid venom proteins are components of a diversified and rapidly evolving host-manipulation system. The hymenopteran literature now provides detailed molecular examples of how venom interferes with melanization, cellular and humoral immunity, host development, metabolism, and behavior. The clearest lesson from this body of work is that similar biological outcomes are routinely achieved by unrelated protein families in different lineages; functional convergence, not strict molecular conservation, is the dominant pattern.

The field is nevertheless constrained by an uneven evidence hierarchy. Omics studies have uncovered a large repertoire of candidate proteins, but direct functional evidence is available for only a minority. The limitation is sharpest outside Hymenoptera. In *D. helophoroides*, host-level immune suppression is clear, yet the identity, tissue source, and mode of action of the relevant effectors remain unresolved.

Future progress will therefore depend on bridging discovery and mechanism. More rigorous functional assays, better anatomical resolution of venom systems, and broader comparative sampling will sharpen both evolutionary interpretation and applied potential, moving parasitoid venomics beyond descriptive catalogs toward a mechanistically grounded and translationally useful discipline.

## Figures and Tables

**Table 1:** Comparison of current strategies for identifying parasitoid venom proteins.

Collection Strategy	Reported Yield (Proteins/Candidates)	Key Advantage	Principal Bias or Limitation	Representative Systems	Reference
Venom reservoir dissection + LC-MS/MS	16–148	Anatomically precise; high-confidence secretion call	Limited by gland size; impractical for small-bodied taxa	*Aphidius ervi*; *Pteromalus puparum*; reservoir fraction of *Habrobracon hebetor*	[15,27,28]
Whole venom apparatus + proteomics	50–>200	Broader coverage, including membrane-bound and duct proteins	Includes non-secretory cell contents; reduced purity	*Cotesia chilonis*; *Habrobracon hebetor*; *Cotesia congregata*	[17,32,37]
Artificial-host collection	25 shared/148 reservoir (partial overlap)	Captures the actually secreted fraction	Misses non-secreted or low-abundance effectors	*Habrobracon hebetor*	[28]
Whole-body extracts of neonate larvae	~50	Feasible when host-attack structures are too small for dissection	Non-venom contamination; no anatomical resolution	*Dastarcus helophoroides*	[19]
Venom-gland transcriptomics only	Hundreds of candidates per species	High throughput; works without a genome	No direct evidence of secretion; homology-based filters miss orphan genes	*Nasonia vitripennis*; *Habrobracon hebetor*; *Gonatopus flavifemur*; *Diversinervus elegans*	[37,45,48,50]
Integrated transcriptomics + proteomics (± genomics)	100–400 high-confidence	Strongest candidate set; enables co-option, convergence, and LGT analyses	Requires paired tissue sampling; higher cost and bioinformatic load	*Chelonus inanitus*; *Toxoneuron nigriceps*; *Pachycrepoideus vindemmiae*; *Leptopilina boulardi/heterotoma* EVs; *Cotesia congregata*	[16,17,24,52,53]

EV, extracellular vesicle; LC-MS/MS, liquid chromatography–tandem mass spectrometry; LGT, lateral gene transfer. Whole-body extracts of neonate larvae are not a generally recommended approach but are forced by small body size; they should be replaced by gland-resolved sampling once the venom apparatus is anatomically identified.

**Table 2:** Selected mechanistically investigated parasitoid venom effectors, venom-induced host responses, and host targets.

Parasitoid Species	Host Species	Venom Protein	Host Target	Key Mechanism/Quantitative Finding	Primary Validation	Reference
*Pteromalus puparum*	*Pieris rapae*	PpS1V (*PpSerpin-1* isoform)	Clip-SPs PrPAP1, PrHP8	SDS-stable RCL complex (stoichiometry ≈ 2.3); blocks proPO activation upstream of PO	Recombinant protein + SDS-PAGE	[59]
*Microplitis mediator*	*Helicoverpa armigera*	MmvSPN-1, MmvSPN-2	Clip-SP HacSP29	Suicide-substrate complexes (SDS-stable ~110 kDa); RNAi de-represses proPO + AMPs	RNAi + biochemistry	[56]
*Cotesia rubecula*	*Pieris rapae*	Vn50 (non-catalytic SPH)	proPAP, PAP, proPO, PO	Full-length Vn50 out-competes host SPHs at proPO interface; individual domains inactive	Domain-mapping + competition	[60]
*Leptopilina boulardi*	*Drosophila* spp.	LbSPNy (54 kDa serpin)	Host hemolymph serine proteases	Classical RCL complex; abundance evolves rapidly under host-specific selection	Recombinant + experimental evolution	[61,62]
*Aphidius ervi*	*Acyrthosiphon pisum*	AeSPH	proPO-activating factor PAF2	RNAi reduces mummification by ~60%	RNAi	[57]
*Aphidius ervi*	*Acyrthosiphon pisum*	AeSPH1, AeSPN1	*ApPPO1* transcription	Selective suppression (not *ApPPO2*); AeSPH1 RNAi lowers mummification by ~55%	RNAi + recombinant	[41]
*Pachycrepoideus vindemmiae*	*Drosophila melanogaster*	PvKazal	Host PO system	Inhibits melanization in *Drosophila melanogaster* by both recombinant injection and transgenic expression	Recombinant injection + *D. melanogaster* transgenic expression	[58]
*Leptopilina boulardi*	*Drosophila* spp.	LbGAP (RhoGAP)	Rac1, Rac2 GTPases	In vitro GAP activity on G12V forms; ISm/ISy virulence difference driven by ~60× abundance, not sequence	Yeast two-hybrid + biochemistry + F1 hybrid	[39]
*Leptopilina heterotoma*	*Drosophila* spp.	Lar	Host lymph gland	Apoptotic lysis within 24 h; RNAi collapses parasitoid emergence 77% → 27%	RNAi	[63]
*Leptopilina boulardi*	*Drosophila* spp.	Warm	Host gut wall	Attaches > 95% of wasp eggs to internal tissues within 4 h (physical protection from encapsulation)	RNAi	[63]
*Microplitis mediator*	*Helicoverpa armigera*	VRF1 (metalloprotease)	NF-κB Dorsal (Cys73/Glu74)	Zymogen cleaved at Arg150-Val151 to release 45 kDa catalytic fragment; suppresses 6 AMPs after *Beauveria bassiana* challenge	Biochemistry + in vivo	[64]
*Microplitis mediator*	*Helicoverpa armigera*	MmGAP1 (RhoGAP)	RhoA, Cdc42	Disrupts hemocyte F-actin; RNAi restores encapsulation in 43.1% of host larvae	RNAi + antibody neutralization	[65]
*Tetrastichus brontispae*	*Brontispa longissima*	Tb4CL4-like (acyl-activating enzyme)	Host hemocytes	Inhibits hemocyte spreading, phagocytosis, and cellular encapsulation	Recombinant protein	[66]
*Pachycrepoideus vindemmiae*	*Drosophila melanogaster*	PvCRT (calreticulin)	Self-encapsulation	Ectopic expression suppresses encapsulation in *D. melanogaster*	*D. melanogaster* transgenic expression	[36]
*Pteromalus puparum*	*Pieris rapae*	Venom-induced Pr-CTL downregulation	Host C-type lectin Pr-CTL	Time- and dose-dependent suppression (peak at 8 h); *Pr-CTL* RNAi lowers encapsulation, phagocytosis, PO, and AMP activity	RNAi + recombinant binding	[67]
*Asobara japonica*	*Drosophila* spp.	IDDF-1, IDDF-2 (DUF4803)	Host imaginal discs	Co-injection yields partial apoptosis + autophagy only; full mitotic arrest requires ~200 additional venom proteins	Recombinant co-injection	[9]
*Asobara japonica*	*Drosophila* spp.	VID-1 to VID-5 (DUF4803 paralogs)	Host imaginal discs	Single-*VID* RNAi collapses disc degradation from 100% (60/60) to 3.3% (2/60); ~26 h pupation delay enables exploitation of 3rd-instar hosts	RNAi	[11]
*Nasonia vitripennis*	*Sarcophaga bullata*	Bulk venom (metalloprotease implicated)	Chitin biosynthesis + Notch-E(spl)-C	100–2072× ↓ cuticular-protein transcripts; 568.7× ↓ peritrophin-A; E(spl)-C ↑ 2.6–6.5× across 9 members	Microarray + transcriptomics	[68]
*Leptopilina* spp.	*Drosophila* spp.	LVL (*Leptopilina*-specific venom lipase)	Host TGs and DGs	pH 6–7 optimum matches parasitization-induced hemolymph acidification; RNAi lowers egg-to-larva hatching by 31.6–54.2%	RNAi + biochemistry	[54]
*Ampulex compressa*	*Periplaneta americana*	Recombinant AcVTk1 (tachykinin)	Host CNS escape threshold	Recombinant peptide recapitulates the short-term elevation of escape threshold	Recombinant + electrophysiology	[44]

AMP, antimicrobial peptide; DG, diacylglycerol; PO, phenoloxidase; proPO, prophenoloxidase; PPO, same as proPO (gene form *ApPPO1*/*ApPPO2*); RCL, reactive-center loop; RhoGAP, Rho GTPase-activating protein; RNAi, RNA interference; SP, serine protease; SPH, serine protease homolog; TG, triacylglycerol.

## Data Availability

No new data were created or analyzed in this study. Data sharing is not applicable to this article.
